# Notes and Comments

**Published:** 1898-11

**Authors:** 


### Notes and Comments.[1]

Dr. Black's Theory.—Dr. Black says that all teeth are equally soft; that the dentine of all teeth is the same in density; that they have the same specific gravity; that a good tooth is no better than a bad tooth; that any filling-material that will save one tooth will save any other, and that there is no choice except on the part of the dentist between lead or tin or gold or amalgam. Well, now, I do not believe that bad teeth are always bad. I do not believe that teeth that are bad in the beginning are bad all through life. I am honest in being mistaken, if I am mistaken, but I believe I have seen changes in the teeth of some of my patients. I call to mind a patient who for ten years suffered from caries of the teeth. He was then leading a sedentary life. He changed his occupation, becoming a railroad man, thus securing plenty of air and exercise. His teeth before required constant care. Now he will go sometimes for five or more years without a single cavity in his teeth. He is an unprofitable patient because his teeth do not require any care. Dr. Black would say that they could not change for the better.—Dr. Edwin T. Darby.

---

Christian Science Helpful in Dental Practice.—The application of Christian Science to the practice of dentistry, I at first thought one of the impossibilities, but with my little understanding I desire to say that it applies better than I could have even hoped

[1] The assistant editor solicits contributions for this department,—new methods, new remedies and formulas, or any short practical note which may prove of value to the practitioner or student. Address 1338 Walnut Street, Philadelphia.

for. It has enabled me to do more work, do it better, and with less fatigue than I ever did before.

It has enabled me to allay the fear of my patients to that extent that I hardly know what it is to have an irritated and troublesome patient in the chair. In the handling of children I operate with almost as much ease as with the ordinary adult, provided I can have the child away from the parents, who generally insist on standing at the chair, forcing the child into the idea that the work is very painful.—Dr. Charles Van Fossen in *Items of Interest.*

---

Concerning Fees.—One of the first things for a patient to learn is that the remuneration for the services of a dentist cannot be understood from the stand-point of merchandising. That is, the services and the fees are not governed by the usual laws of trade. The exchange of values does not constitute a barter or sale. Value is given and money is received by the dentist, but the exchange is unequal. The services, involving, as they do, the comfort, the health, and at times in no slight degree the life of the patient, are inestimable, therefore the money received does not pay for them.—Dr. L. Ashley Faught.

Not the least valuable item on the inventory of the dentist's stock is his precious moments,—sixty of which make an hour,—for every one of which he should receive compensation when reserved for patrons and the appointment is broken without reasonable notice,—and reasonable notice is not one minute or one hour.

The fees charged should, therefore, be reasonably commensurate with the foregoing facts and the substantial character of the service rendered.—Dr. A. B. Freeman.

---

Alcohol as a Disinfectant.—Recent researches seem to show that absolute alcohol is devoid of all disinfectant properties. Proof spirit (fifty per cent.) gives more tangible results in this direction than either stronger or weaker solutions. Antiseptic substances, which in aqueous solution are more or less active germicides, entirely lose this property when dissolved in strong alcohol. But on the other hand corrosive sublimate, carbolic acid, lysol, and thymol dissolved in a fifty-per-cent. solution alcohol disinfect better than aqueous solutions of the same strength.—*Medical Press and Circular.*

Salivary Calculus in the Parotid Duct.—Dr. T. E. Constant, in the *Journal of the British Association*, reports a case where the accumulation of calculus in the parotid duct caused symptoms simulating alveolar abscess. The patient called upon him to have a tooth extracted. The left cheek was considerably swollen, which swelling had commenced three days previous. He says the cheek was both painful and tender, but the skin, although tense, was not reddened. The swelling was most prominent in the centre of the cheek. Pressure over the region of the left parotid caused pain. The patient was very nervous, and examination of the mouth in consequence was somewhat difficult. The patient said the trouble "commenced with pain in the last tooth in the upper jaw,"—viz., the left upper second molar. When that tooth was gently tapped she complained of pain, although it was apparently a sound tooth. She said it had been very painful in eating during the last three days. Careful examination, however, enabled me to assure myself that the swelling in the cheek was not connected with either the upper or the lower jaw, and I noted a grayish appearance at the most prominent part of the swelling in the mucous surface of the cheek.

At first I thought it was a commencing slough, but subsequently found it to be due to something white showing through the mucous membrane. With some difficulty I passed a probe into the parotid duct and was able to dislodge a small calculus, about the size of a one-grain cocaine tabloid and very much of that shape. I saw the patient again two days later, when the swelling had completely subsided, and the mouth "felt perfectly comfortable."

---

Board of Censors in Place of Examining Boards.—From Dr. B. F. Arrington's recent contribution to this journal we take the following:

"In place of examining boards I will suggest that each State create a board of censors, to whom shall be reported any and all cases of malpractice or improper professional conduct, and before whom the accused may and shall confront his accusers, and, if convicted, shall be suspended or barred the privilege to practise in the State in which the offence was committed; and the secretary of the board of censors shall be required to report proceedings and ruling, with seal of office, to every other board in the several States, who shall place on file and furnish copy to secretary of State dental association or society, to be read at ensuing meeting. By such a procedure the unworthy could be rightly dealt with, the public protected more securely, and the profession sustained better than by the present provision and arrangement of examining boards."

ADDITIONAL DENTAL COLLEGES NOT AN EDUCATIONAL NECESSITY.—In an essay presented at the forty-eighth annual meeting of the American Medical Association, Dr. Richard Grady said that the dental schools are not being formed now from an educational necessity.

Two impulses control this matter,—1, personal ambition to have a position in and be connected with a dental school, for the prominence it is supposed to give; and, 2, a purely commercial spirit on the part of medical schools. Over 60 per cent. of our schools are appendages to medical institutions; and nearly every dental school started in these later years has been under this outside influence.

